# Clinical and immunopathological evaluation and its comparison with IHC consensus molecular subtypes of colorectal cancer

**DOI:** 10.1038/s41598-025-04962-w

**Published:** 2025-07-01

**Authors:** Eduardo Feliciangeli, Ana Albaladejo-González, José García-Rodríguez, Antonio Lázaro-Sánchez, Rosanna Borg, Paola Pimentel-Cáceres, Diego Soriano-Polo, Edith Rodríguez-Braun, José Balsalobre-Yago, María José Martínez-Ortiz, Sofía Wikström-Fernández, Andrés Murillo-Herrera, Teresa García-García, José García-Solano, Pablo Conesa-Zamora, Ginés Luengo-Gil

**Affiliations:** 1 Medical Oncology Department, Hospital General Universitario Santa Lucía, Cartagena, Spain; 2 Group of Molecular Pathology and Pharmacogenetics, Instituto Murciano de Investigación Biosanitaria (IMIB), Hospital Universitario Santa Lucía, Cartagena, Spain; 3 Health Sciences Faculty, Universidad Católica de Murcia (UCAM), Guadalupe, Spain; 4 Laboratory Medicine and Pathology Department, Hospital General Universitario Santa Lucía, Cartagena, Spain; 5 Van Hall Larenstein University of Applied Sciences, Agora 1, P.O. Box 1528, 8901 BV Leeuwarden, The Netherlands; 6 Medical Oncology Department, Hospital General Universitario Morales Meseguer, Murcia, Spain

**Keywords:** Colon cancer, Rectal cancer, Cancer

## Abstract

This study examined the prognostic significance of immunoscore within consensus molecular subtypes (CMS), tumour budding (TB), and macrophage infiltration in colorectal cancer (CRC). A retrospective series was analysed using immunohistochemical staining, immunoscore evaluation, and CMS classification, according to established methods. Among the 255 patients with CRC, 34.9% had stage III disease. The CMS classification showed a predominance of CMS2/3 (69.4%), with relapse occurring across all the subtypes. Low immunoscore was frequent in conventional and serrated adenocarcinomas, as well as in CMS2/3, whereas MSI-H correlated with intermediate-high immunoscore. TB was prevalent in relapsed patients, particularly in CMS2/3 and CMS4, and was linked to serrated histology. TB of ≥ 20 foci was correlated with hepatic metastases. CD163+ macrophage infiltration was common in CMS1 and CMS2/3, and was associated with high immune scores. TB influenced overall and relapse-free survival, whereas CMS affected the overall survival. Immunoscore were not associated with survival. The associations identified between CMS subtypes and clinical as well as pathological characteristics enhance the understanding of CMS behaviour in clinical practice.

**Supplementary Information:**

The online version contains supplementary material available at 10.1038/s41598-025-04962-w.

## Introduction

Colorectal cancer (CRC) is a prevalent malignancy worldwide, with an estimated 1,931,590 new cases in 2020, and a projected increase of 3.2 million by 2040^1^. Despite advancements in treatment and screening, CRC mortality remains a significant public health concern, with 0.9 million deaths globally in 2020^1^. The 5-year survival rate varies depending on the disease stage, ranging from 91% for localised disease to 13% for metastatic disease (American Cancer Society. Key Statistics for Colorectal Cancer. Updated 2025).

No studies have explicitly investigated the relationship between molecular subtypes, histology, tumour budding, immunoscore, and macrophage infiltration in colon cancer. Such research could further enhance our understanding of this complex disease, not only in terms of prognosis but also in predicting treatment response.

It is well known that CRC is a heterogeneous disease with variations in morphological subtypes and developmental sequences, including genetic instability at the nucleotide sequence (MSI-H) or chromosomal (CIN) level^2–4^. Sporadic MSI-H CRCs differ from Lynch syndrome-associated CRCs due to extensive DNA methylation and *BRAF* mutations^5,6^. Most CRCs are microsatellite stable (MSS), with only 10% showing classic *APC*, *KRAS*, and *TP53* mutation genotypes^5,7^. Weisenberger et al.^8^ classified CRC into CpG-island methylator phenotype (CIMP)-High (CIMP1), CIMP-Low (CIMP2), and CIMP-negative groups based on mutations and clinical characteristics. In 2007, Jass et al.^2^ introduced a molecular classification system using CIMP, MSI, *KRAS*, *BRAF*, and *MGMT* methylation, resulting in five subtypes. However, Zlobec et al.^9^ in 2012 raised concerns regarding the reproducibility and definition of CIMP in this classification. By 2015, existing classifications were confusing and inconsistent, with three to six molecular subtypes identified based on gene expression, but showing only superficial similarities. To address this, the Colorectal Cancer subtyping consortium (CRCSC) was developed to evaluate subtype patterns in existing CRC subtyping algorithms based on gene expression^10^. CRCSC established a gene expression-based classification with four subtypes:

- CMS1 (MSI-immune, 14%): High mutation rate, immune infiltration, *BRAF* mutations, and poor survival after relapse
- CMS2 (canonical, 37%): MSS tumors, CIN, WNT/MYC pathway activation, and better survival after relapse
- CMS3 (metabolic, 13%): Low CIN, high CIMP presence, *KRAS*, and *PI3K* mutations
- CMS4 (mesenchymal, 23%): CIN tumours, mesenchymal features, and poor prognosis.

The molecular subtypes provide valuable insights into CRC biology. However, they do not fully capture the complexity of tumour-host interactions or invasive behaviour. Additionally, they are not commonly used in routine clinical settings. Two key factors that complement molecular subtyping and offer additional prognostic information are immunoscore and tumour budding. Immunoscore quantifies the immune response within the tumour microenvironment, whereas tumour budding reflects the invasive potential of the tumour. Investigating these factors in relation to molecular subtypes may provide a more comprehensive understanding of CRC progression and treatment responses.

The immunoscore, developed by Galon et al.^11^, evaluates the density and location of CD3+ and CD8+ tumor-infiltrating lymphocytes^11,12^. This metric has shown strong prognostic value in CRC, often outperforming the traditional TNM staging. A high immunoscore correlates with better survival, and has been validated as an independent predictor of recurrence and survival. This marker can enhance risk stratification and guide personalized immunotherapy^13^. Understanding how the immunoscore relates to molecular subtypes could reveal important insights into the interplay between tumour biology and the immune response.

Immunoscore can be integrated into clinical decision algorithms along with other molecular and clinical markers to provide a more comprehensive prognosis and optimise the treatment. Combining immunological, genetic, and clinical data promises to revolutionise CRC treatment and improve patient survival and quality of life. Despite the established role of molecular subtypes and immunoscore in predicting CRC outcomes, their interplay and combined prognostic value remains unclear.

Macrophages are highly plastic cells capable of responding to and adapting to external stimuli, and they play a crucial role in the tumour microenvironment. Two primary phenotypes were identified: M1 and M2^14^. M1 macrophages, induced by factors such as lipopolysaccharide (LPS) and interferon-gamma (IFN-γ), exhibit pro-inflammatory properties and are characterised by a high capacity for antigen presentation and production of cytokines such as Interleukin-1 beta (IL1-β) and tumour necrosis factor (TNF)^14,15^. In contrast, M2 macrophages, induced by IL4 and IL13, are associated with anti-inflammatory functions such as promoting angiogenesis and tumour invasion^14,15^.

Studies have demonstrated that Tumour-associated macrophages (TAMs), particularly those of the M2 phenotype, are often associated with poor prognosis in various cancer types, including colorectal cancer^16–18^. However, this evidence is not entirely consistent. Some studies suggest that a higher presence of macrophages correlates with more advanced tumour stages and poorer outcomes, whereas others indicate that TAMs may be linked to improved survival in colorectal cancer and a lower likelihood of liver metastasis. This discrepancy suggests that the balance between M1 and M2 macrophages is critical for patient outcome.

Inactive macrophages (M0) typically exhibit a slightly elongated morphology, while M1 macrophages are more rounded and M2 macrophages are elongated^19^. These morphological changes are dynamic and depend on the extracellular matrix, which is influenced by the surrounding microenvironment^20^. Additionally, it has been observed that M2 macrophages with rounded morphology can promote neovascularization, indicating that this shape is not exclusive to a specific phenotype^21^.

Tumour budding, defined as the presence of single tumour cells or small clusters (up to four cells) at the invasive front, is another important prognostic factor for CRC^22,23^. It is thought to represent the morphological manifestation of epithelial-mesenchymal transition, a key process in tumour invasion and metastasis. The relationship between tumour budding and molecular subtypes remains poorly understood, presenting an opportunity to link tumour biology with invasive behaviour. Evidence suggests that tumour buds may exhibit characteristics of cells undergoing epithelial-mesenchymal transition (EMT), indicating that these cells possess enhanced invasive and migratory capabilities. Increasing data indicate that tumour budding is an unfavourable prognostic marker^24^.

In patients with pT1 colon cancer, intermediate and high tumour budding is associated with lymph node involvement, which guides the decision to pursue additional radical surgery following endoscopic resection^25^. In stage II colon cancer, a high tumour-budding category is considered a poor prognostic indicator that should be factored into decisions regarding adjuvant therapy^26,27^. However, the prognostic significance of tumour budding in stage III colon cancer has primarily been examined in small retrospective studies. In a post-hoc analysis of the IDEA-France phase III study, researchers evaluated the prognostic significance of tumour budding using the International Tumour Budding Consensus Conference (ITBCC) 2016 criteria in patients with stage III colon cancer, demonstrating its independent prognostic value for both disease-free survival (DFS) and overall survival (OS)^22,28^.

Colorectal cancer (CRC) is a clinically and biologically diverse entity that requires precise characterisation and treatment strategies. The consensus molecular subtype (CMS) Classification of CRC is one of the most promising areas of research in this regard; however, it is expensive and not available in every centre. This study aimed to evaluate the molecular subtypes using immunohistochemistry and a validated online classifier (https://crcclassifier.shinyapps.io/appTesting/). This approach allows for a deeper understanding of the molecular heterogeneity of CRC, and the study of its association with various clinical and biological aspects, such as clinicopathological characteristics, macrophage infiltration, and immunoscore, could potentially shed more light on this disease.

Our study aimed to explore the link between immunoscore and CRC heterogeneity, as defined by CMS, in predicting disease progression and treatment response.

By examining immunoscore and tumour budding in the context of CRC molecular subtypes, this study aimed to:

1. Evaluate the distribution of immunoscore and tumour budding across different molecular subtypes.
2. Assess the combined prognostic value of these factors.
3. Explore correlations between molecular subtypes, immune responses, and invasive behaviour.
4. Identify subgroups of patients who might benefit from tailored treatment approaches based on integrated assessments.

## Results

### Patient characteristics and molecular subtypes

A cohort of 255 colorectal cancer patients was diagnosed between 2006 and 2020, with an average age of 63.5 years (30–85), including 57.6% men. The ethnic breakdown was as follows: 60.0% Caucasian, 25.0% Hispanic, and 15.0% other. Of the 235 patients with molecular subtype data, 20.4% had CMS1, 69.4% had CMS2/3, and 10.2% had CMS4. The disease stages were 1.2% stage 0, 10.2% stage I, 34.9% stage II, 34.9%; stage III, and 18.8% stage IV. Adjuvant treatment was administered to 40.8% of the patients, with capecitabine plus oxaliplatin adjuvant chemotherapy (CAPOX) being the predominant regimen (Table 1). The associations between clinicopathological variables and CMS are shown in Table 2.

**Table 1:** Clinical and pathological characteristics.

Characteristic	N	%
*N*	255	100%
Age (median; min–max)	70.0 (18–92)	
Sex		
Male	147	57.6
Female	108	42.4
Clinical stage		
0	3	1.2
I	26	10.2
IIA	78	30.6
IIB	10	3.9
IIC	1	0.4
IIIA	4	1.6
IIIB	59	23.1
IIIC	26	10.2
IV	48	18.8
Clinical stage of primary tumor		
T1	10	3.9
T2	21	8.2
T3	155	60.8
T4	65	25.5
Not avalilable	4	1.6
Lymph node clinical stage		
N0	126	49.4
N1	72	28.2
N2	54	21.2
Histological type		
Conventional	88	34.5
Cribiform-comedo	42	16.5
MSI	42	16.5
Mucinous	20	7.8
Serrated	63	24.7
Histological tumor grade		
G1	66	25.9
G2	160	62.7
G3	28	11
Not available	1	0.4
Sidedness		
Left	142	55.7
Right	113	44.3
Colon or rectum		
Colon	207	81.2
Rectum	48	18.8
Lymphatic invasion		
No	171	67.1
Yes	80	31.4
Not available	4	1.6
Vascular invasion		
No	212	83.1
Yes	39	15.3
Not available	4	1.6
Perineural invasion		
No	198	77.6
Yes	53	20.8
Not available	4	1.6
Tumor budding		
No	99	38.8
< 10 foci	97	38
10–19 foci	34	13.3
> = 20 foci	18	7.1
Not Available	7	2.7
Consensus molecular subtypes		
CMS1	48	18.8
CMS2/3	163	63.9
CMS4	24	9.4
Not available	20	7.8
Immunoscore (2 categories)		
Low	87	34.1
Intermediate/High	21	8.2
Not available	147	57.6
Treatment (localized disease)		
Adjuvant	104	40.8
Not indicated	151	59.2
Relapse in localized disease (n = 206)		
Yes	40	19.4
No	166	80.6

**Table 2:** Association of clinical and pathological variables with molecular subtype and immunoscore.

	Molecular subtype		Immunoscore		Tumor budding	
	n	*p*-value	n	*p*-value	n	*p*-value
Age at diagnosis*						
Median and interquartile range: 68.8 years [IQR 61–77]	CMS1: 48	0.401	Low: 87	0.988	No: 99	0.476
	CMS2-3: 163		Int-High: 21		< 10: 97	
	CMS4: 24				10–19: 34	
					> = 20: 18	
Sex						
Male	CMS1: 19	0.003	Low: 52	0.858	No: 57	0.983
	CMS2-3: 105		Int-High: 13		< 10: 58	
	CMS4: 10				10–19: 20	
					> = 20: 10	
Female	CMS1: 29		Low: 35		No: 42	
	CMS2-3: 58		Int-High: 8		< 10: 39	
	CMS4: 14				10–19: 14	
					> = 20: 8	
ECOG						
1	CMS1: 34	0.908	Low: 61	0.767	No: 75	0.304
	CMS2-3: 123		Int-High: 13		< 10: 69	
	CMS4: 16				10–19: 28	
					> = 20: 11	
2	CMS1: 10		Low: 17		No: 17	
	CMS2-3: 27		Int-High: 5		< 10: 20	
	CMS4: 5				10–19: 3	
					> = 20: 5	
3	CMS1: 4		Low: 8		No: 7	
	CMS2-3: 12		Int-High: 3		< 10: 8	
	CMS4: 3				10–19: 2	
					> = 20: 2	
4	CMS1: 0		Low: 1		No: 0	
	CMS2-3: 1		Int-High: 0		< 10: 0	
	CMS4: 0				10–19: 1	
					> = 20: 0	
Clinical stage						
I	CMS1: 4	0.039	Low: 13	0.459	No: 16	< 0.001
	CMS2-3: 17		Int-High: 1		< 10: 9	
	CMS4: 1				10–19: 1	
					> = 20: 0	
II	CMS1: 19		Low: 29		No: 41	
	CMS2-3: 60		Int-High: 6		< 10: 34	
	CMS4: 7				10–19: 9	
					> = 20: 1	
III	CMS1: 17		Low: 32		No: 26	
	CMS2-3: 57		Int-High: 10		< 10: 35	
	CMS4: 6				10–19: 18	
					> = 20: 7	
IV	CMS1: 8		Low: 12		No: 13	
	CMS2-3: 26		Int-High: 4		< 10: 19	
	CMS4: 10				10–19: 6	
					> = 20: 10	
Sidedness						
Left	CMS1: 6	< 0.001	Low: 47	0.358	No: 45	0.021
	CMS2-3: 105		Int-High: 9		< 10: 64	
	CMS4: 16				10–19: 17	
					> = 20: 12	
Right	CMS1: 42		Low: 40		No: 54	
	CMS2-3: 58		Int-High: 12		< 10: 33	
	CMS4: 8				10–19: 17	
					> = 20: 6	
Colon or rectum						
Colon	CMS1: 47	0.001	Low: 72	0.74	No: 76	0.13
	CMS2-3: 129		Int-High: 18		< 10: 79	
	CMS4: 19				10–19: 31	
					> = 20: 17	
Rectum	CMS1: 1		Low: 15		No: 23	
	CMS2-3: 34		Int-High: 3		< 10: 18	
	CMS4: 5				10–19: 3	
					> = 20: 1	
Polyps						
Yes	CMS1: 35	0.001	Low: 52	0.826	No: 55	0.532
	CMS2-3: 77		Int-High: 12		< 10: 50	
	CMS4: 8				10–19: 14	
					> = 20: 10	
No	CMS1: 13		Low: 35		No: 44	
	CMS2-3: 85		Int-High: 9		< 10: 47	
	CMS4: 16				10–19: 20	
					> = 20: 8	
Carcino-embryonic antigen, CEA (presurgical)						
= < 5	CMS1: 24	0.704	Low: 44	0.685	No: 52	0.038
	CMS2-3: 89		Int-High: 12		< 10: 56	
	CMS4: 14				10–19: 21	
					> = 20: 4	
> 5	CMS1: 11		Low: 19		No: 18	
	CMS2-3: 30		Int-High: 4		< 10: 17	
	CMS4: 4				10–19: 6	
					> = 20: 7	
Histological grade						
1	CMS1: 5	< 0.001	Low: 24	0.765	No: 27	0.08
	CMS2-3: 50		Int-High: 5		< 10: 26	
	CMS4: 4				10–19: 8	
					> = 20: 4	
2	CMS1: 21		Low: 55		No: 54	
	CMS2-3: 108		Int-High: 15		< 10: 66	
	CMS4: 18				10–19: 24	
					> = 20: 10	
3	CMS1: 22		Low: 7		No: 17	
	CMS2-3: 4		Int-High: 1		< 10: 5	
	CMS4: 2				10–19: 2	
					> = 20: 4	
MSI						
Yes	CMS1: 46	< 0.001	Low: 8	0.011	No: 37	< 0.001
	CMS2-3: 0		Int-High: 8		< 10: 5	
	CMS4: 0				10–19: 1	
					> = 20: 3	
No	CMS1: 0		Low: 77		No: 61	
	CMS2-3: 163		Int-High: 13		< 10: 92	
	CMS4: 24				10–19: 32	
					> = 20: 15	
CIMP						
No	CMS1: 7	0.075	Low: 12	0.864	No: 10	0.134
	CMS2-3: 21		Int-High: 3		< 10: 13	
	CMS4: 1				10–19: 3	
					> = 20: 6	
Low	CMS1: 3		Low: 9		No: 9	
	CMS2-3: 19		Int-High: 1		< 10: 10	
	CMS4: 3				10–19: 9	
					> = 20: 1	
High	CMS1: 5		Low: 4		No: 4	
	CMS2-3: 4		Int-High: 1		< 10: 5	
	CMS4: 0				10–19: 1	
					> = 20: 0	
*BRAF*						
Native	CMS1: 32	< 0.001	Low: 80	0.691	No: 76	0.063
	CMS2-3: 155		Int-High: 19		< 10: 92	
	CMS4: 23				10–19: 33	
					> = 20: 17	
Mutated	CMS1: 13		Low: 6		No: 13	
	CMS2-3: 6		Int-High: 2		< 10: 5	
	CMS4: 1				10–19: 1	
					> = 20: 1	
*KRAS*						
Native	CMS1: 29	0.107	Low: 36	0.207	No: 55	0.005
	CMS2-3: 76		Int-High: 12		< 10: 46	
	CMS4: 11				10–19: 13	
					> = 20: 4	
Mutated	CMS1: 16		Low: 50		No: 34	
	CMS2-3: 85		Int-High: 9		< 10: 51	
	CMS4: 13				10–19: 21	
					> = 20: 14	
*NRAS*						
Native	CMS1: 40	0.845	Low: 76	0.366	No: 74	0.288
	CMS2-3: 138		Int-High: 17		< 10: 84	
	CMS4: 21				10–19: 30	
					> = 20: 18	
Mutated	CMS1: 5		Low: 10		No: 15	
	CMS2-3: 23		Int-High: 4		< 10: 13	
	CMS4: 3				10–19: 4	
					> = 20: 0	
CDX2						
Positive	CMS1: 27	< 0.001	Low: 8	0.436	No: 72	0.007
	CMS2-3: 155		Int-High: 3		< 10: 89	
	CMS4: 21				10–19: 31	
					> = 20: 12	
Negative	CMS1: 13		Low: 75		No: 13	
	CMS2-3: 4		Int-High: 16		< 10: 3	
	CMS4: 1				10–19: 0	
					> = 20: 2	
Tumor growth pattern						
Infiltrative	CMS1: 8	< 0.001	Low: 31	0.15	No: 8	< 0.001
	CMS2-3: 64		Int-High: 4		< 10: 47	
	CMS4: 14				10–19: 24	
					> = 20: 16	
Expansive	CMS1: 40		Low: 53		No: 88	
	CMS2-3: 91		Int-High: 16		< 10: 50	
	CMS4: 10				10–19: 10	
					> = 20: 2	
Tumor budding						
< 10	CMS1: 5	< 0.001	Low: 33	0.079	-	
	CMS2-3: 80		Int-High: 10			
	CMS4: 7					
Oct-19	CMS1: 3		Low: 6			
	CMS2-3: 10		Int-High: 1			
	CMS4: 2					
> = 20	CMS1: 2		Low: 14			
	CMS2-3: 21		Int-High: 0			
	CMS4: 7					
Histology						
Conventional	CMS1: 0	< 0.001	Low: 32	0.025	No: 28	< 0.001
	CMS2-3: 74		Int-High: 6		< 10: 34	
	CMS4: 8				10–19: 15	
					> = 20: 4	
Serrated	CMS1: 3		Low: 26		No: 11	
	CMS2-3: 48		Int-High: 2		< 10: 30	
	CMS4: 4				10–19: 11	
					> = 20: 11	
Comedo-cribiform	CMS1: 0		Low: 11		No: 12	
	CMS2-3: 31		Int-High: 4		< 10: 23	
	CMS4: 7				10–19: 6	
					> = 20: 1	
Mucinous	CMS1: 3		Low: 8		No: 14	
	CMS2-3: 10		Int-High: 1		< 10: 6	
	CMS4: 5				10–19: 0	
					> = 20: 0	
Serrated adenocarcinoma						
Yes	CMS1: 3	0.001	Low: 26	0.056	No: 11	< 0.001
	CMS2-3: 48		Int-High: 2		< 10: 30	
	CMS4: 4				10–19: 11	
					> = 20: 11	
No	CMS1: 45		Low: 61		No: 88	
	CMS2-3: 115		Int-High: 19		< 10: 67	
	CMS4: 20				10–19: 23	
					> = 20: 7	
Vascular infiltration						
Yes	CMS1: 10	0.481	Low: 10	0.738	No: 6	< 0.001
	CMS2-3: 23		Int-High: 3		< 10: 13	
	CMS4: 5				10–19: 9	
					> = 20: 10	
No	CMS1: 38		Low: 76		No: 93	
	CMS2-3: 136		Int-High: 18		< 10: 84	
	CMS4: 19				10–19: 25	
					> = 20: 8	
Lymphatic infiltration						
Yes	CMS1: 17	0.252	Low: 26	0.561	No: 12	< 0.001
	CMS2-3: 47		Int-High: 5		< 10: 33	
	CMS4: 11				10–19: 19	
					> = 20: 15	
No	CMS1: 31		Low: 60		No: 87	
	CMS2-3: 112		Int-High: 16		< 10: 64	
	CMS4: 13				10–19: 15	
					> = 20: 3	
Perineural infiltration						
Yes	CMS1: 11	0.818	Low: 14	0.823	No: 8	< 0.001
	CMS2-3: 32		Int-High: 3		< 10: 21	
	CMS4: 6				10–19: 11	
					> = 20: 12	
No	CMS1: 37		Low: 72		No: 91	
	CMS2-3: 127		Int-High: 18		< 10: 76	
	CMS4: 18				10–19: 23	
					> = 20: 6	
Macrophages (CD163 spindle-shape)						
Yes	CMS1: 20	0.073	Low: 58	0.029	No: 47	0.347
	CMS2-3: 95		Int-High: 18		< 10: 55	
	CMS4: 11				10–19: 16	
					> = 20: 6	
No	CMS1: 3		Low: 10		No: 10	
	CMS2-3: 14		Int-High: 0		< 10: 9	
	CMS4: 7				10–19:6	
					> = 20: 0	
Macrophages (CD163 round)						
Yes	CMS1: 18	0.002	Low: 26	0.015	No: 27	0.349
	CMS2-3: 43		Int-High: 12		< 10: 31	
	CMS4: 5				10–19: 6	
					> = 20: 2	
No	CMS1: 6		Low: 42		No: 31	
	CMS2-3: 67		Int-High: 8		< 10: 34	
	CMS4: 13				10–19: 16	
					> = 20: 4	
Macrophages (CD163 total)						
Positive	CMS1: 20	0.027	Low: 61	0.156	No: 47	0.162
	CMS2-3: 98		Int-High: 18		< 10: 59	
	CMS4: 12				10–19: 17	
					> = 20: 6	
Negative	CMS1: 3		Low: 7		No: 10	
	CMS2-3: 11		Int-High: 0		< 10: 5	
	CMS4: 6				10–19: 5	
					> = 20: 0	
Adjuvant treatment						
Yes	CMS1: 20	0.933	Low: 35	0.858	No: 38	0.039
	CMS2-3: 64		Int-High: 8		< 10: 33	
	CMS4: 9				10–19: 21	
					> = 20: 8	
No	CMS1: 28		Low: 52		No: 61	
	CMS2-3: 99		Int-High: 13		< 10: 64	
	CMS4: 15				10–19: 13	
					> = 20: 10	
Metastatic chemotherapy regimen						
Qx alone	CMS1: 2	0.47	Low: 2	0.492	No: 2	0.722
	CMS2-3: 2		Int-High: 1		< 10: 2	
	CMS4: 0				10–19:0	
					> = 20: 1	
Qx + antiEGFR	CMS1: 3		Low: 7		No: 8	
	CMS2-3: 15		Int-High: 1		< 10: 6	
	CMS4: 2				10–19: 5	
					> = 20: 2	
Qx + antiangiogenic	CMS1: 4		Low: 3		No: 6	
	CMS2-3: 17		Int-High: 2		< 10: 11	
	CMS4: 4				10–19: 7	
					> = 20: 5	
Number of metastatic locations at diagnosis						
Non-regional nodes	CMS1: 2	0.006	Low: 1	0.398		0.216
	CMS2-3: 1		Int-High: 0			
	CMS4: 0					
Hepatic	CMS1: 1		Low: 7		No: 5	
	CMS2-3: 16		Int-High: 1		< 10: 13	
	CMS4: 3				10–19: 2	
					> = 20: 4	
Peritoneal	CMS1: 4		Low: 4		No: 5	
	CMS2-3: 5		Int-High: 3		< 10: 3	
	CMS4: 8				10–19: 5	
					> = 20: 4	
Lung	CMS1: 1		Low: 1		No: 1	
	CMS2-3: 4		Int-High: 0		< 10: 2	
	CMS4: 0				10–19: 0	
					> = 20: 2	
Number of metastatic locations at progression						
Non-regional nodes						
Hepatic	CMS1: 4	0.005	Low: 3	0.606	No: 3	0.048
	CMS2-3: 1		Int-High: 0		< 10: 10	
	CMS4: 0				10–19: 5	
					> = 20: 6	
Peritoneal	CMS1: 1		Low: 5		No: 9	
	CMS2-3: 16		Int-High: 2		< 10: 6	
	CMS4: 3				10–19: 5	
					> = 20: 1	
Lung	CMS1: 6		Low: 5		No: 4	
	CMS2-3: 8		Int-High: 2		< 10: 9	
	CMS4: 5				10–19: 3	
					> = 20: 0	
Bone	CMS1: 1		Low: 8		No: 1	
	CMS2-3: 13		Int-High: 1		< 10: 0	
	CMS4: 1				10–19: 3	
					> = 20: 1	
Distant nodal	CMS1: 2		Low: 2		No: 3	
	CMS2-3: 2		Int-High: 1		< 10: 0	
	CMS4: 1				10–19: 1	
					> = 20: 1	
Immunoscore						
Low	CMS1: 10	0.015	-		No: 31	0.244
	CMS2-3: 67				< 10: 33	
	CMS4: 8				10–19: 14	
					> = 20: 6	
Intermediate-high	CMS1: 8				No: 9	
	CMS2-3: 12				< 10: 10	
	CMS4: 1				10–19: 0	
					> = 20: 1	

The reference categories for each variable are as follows: for clinical stage, Stage I; for sidedness, left-sided tumors; for histological grade, G1 (well-differentiated); for tumor budding, no tumor budding; for immunoscore, low immunoscore; for adjuvant treatment, no adjuvant treatment; and for molecular subtypes, CMS1. All *p*-values represent results from chi-square tests or contingency tables unless specified otherwise. *Kruskal–Wallis test.

### Immunoscore and its associations

Among the 106 cases with complete CMS and Immunoscore data, a significant association was observed between CMS class and the level of immune infiltration (χ^2^, p = 0.015). Intermediate-to-high Immunoscore (I2–I4) was observed in 44.4% of CMS1 tumours, compared to 15.2% of CMS2/3 and 11.1% of CMS4 tumours. Conversely, low Immunoscore (I0–I1) predominated across all subtypes, being present in 55.6% of CMS1, 84.8% of CMS2/3, and 88.9% of CMS4 tumours. These findings highlight the relatively more immunogenic phenotype of CMS1 and are consistent with prior evidence linking this subtype to active tumour immune engagement. The full distribution of data is provided in Supplementary Table S1.

Among patients with localised disease at diagnosis (n = 206), 40 experienced relapses, representing 19.4% of the cohort. The distribution of relapses by consensus molecular subtype (CMS) was as follows: CMS1 (8/48; 16.7%), CMS2/3 (23/163; 14.1%), and CMS4 (4/24; 16.7%), while 5 patients could not be evaluated for CMS. Immunoscore assessment was available for 17 out of the 40 relapsed patients, with 15 (88.2%) showing a low Immunoscore and 2 (11.8%) presenting an intermediate-to-high Immunoscore. The remaining relapsed patients were not evaluable for immunoscore. Table 2 shows the associations between clinicopathological variables and immunoscore.

### Tumor budding patterns and correlations

Among the relapsed patients, 12 had TB < 10 foci, 12 had 10–19 foci, 4 had TB ≥ 20 foci, and 10 had a serrated histology. Most CMS1 patients had no TB, whereas CMS2/3 subtypes and CMS4 were predominantly TB-positive. CMS2/3 (71.15%) and CMS4 (66.66%) had the highest prevalence of TB. Immunoscore and TB showed no significant relationships. Serrated (82.5%, TB-positive), comedo-cribriform (71.4%), and conventional (65.4%) histology were mainly associated with TB. TB was related to relapses in localised disease, and 28 of the 40 relapsed patients were TB positive. Serrated histology was significantly associated with CMS2/3 tumours (29.4%). Among the 255 patients, 39 (15.3%) had rectal tumours. Tumour budding was associated with specific metastatic sites, particularly hepatic involvement. Data on tumour location were not used as stratification variables in this analysis.

### Metastatic patterns and disease progression

CMS1 (24 patients, 12 with peritoneal metastasis), CMS2/3 (39 patients, 24 with hepatic metastasis), and CMS4 (11 patients, 8 with peritoneal metastasis) were associated with peritoneal (50.0%), hepatic (61.5%), and peritoneal (72.7%) metastases at diagnosis, respectively. Among the 14 CMS1 patients with progression, 6 (42.9%) had peritoneal progression. In the CMS2/3 group, 40.0% (16/40) of patients showed hepatic progression, while in the CMS4 group, 15 patients progressed, with 50.0% (8/15) showing peritoneal progression and 33.3% (5/15) showing hepatic progression. High TB (≥ 20 foci) was correlated with hepatic progression (66.7%), 10–19 foci with both hepatic and peritoneal progression (29.4%), and < 10 foci with hepatic (40.0%), pulmonary (36.0%), and peritoneal (24.0%) progression. The absence of TB was linked to peritoneal (45.0%) and pulmonary (20.0%) disease progressions.

### Macrophage infiltration and immune microenvironment

**Fig. 1:**
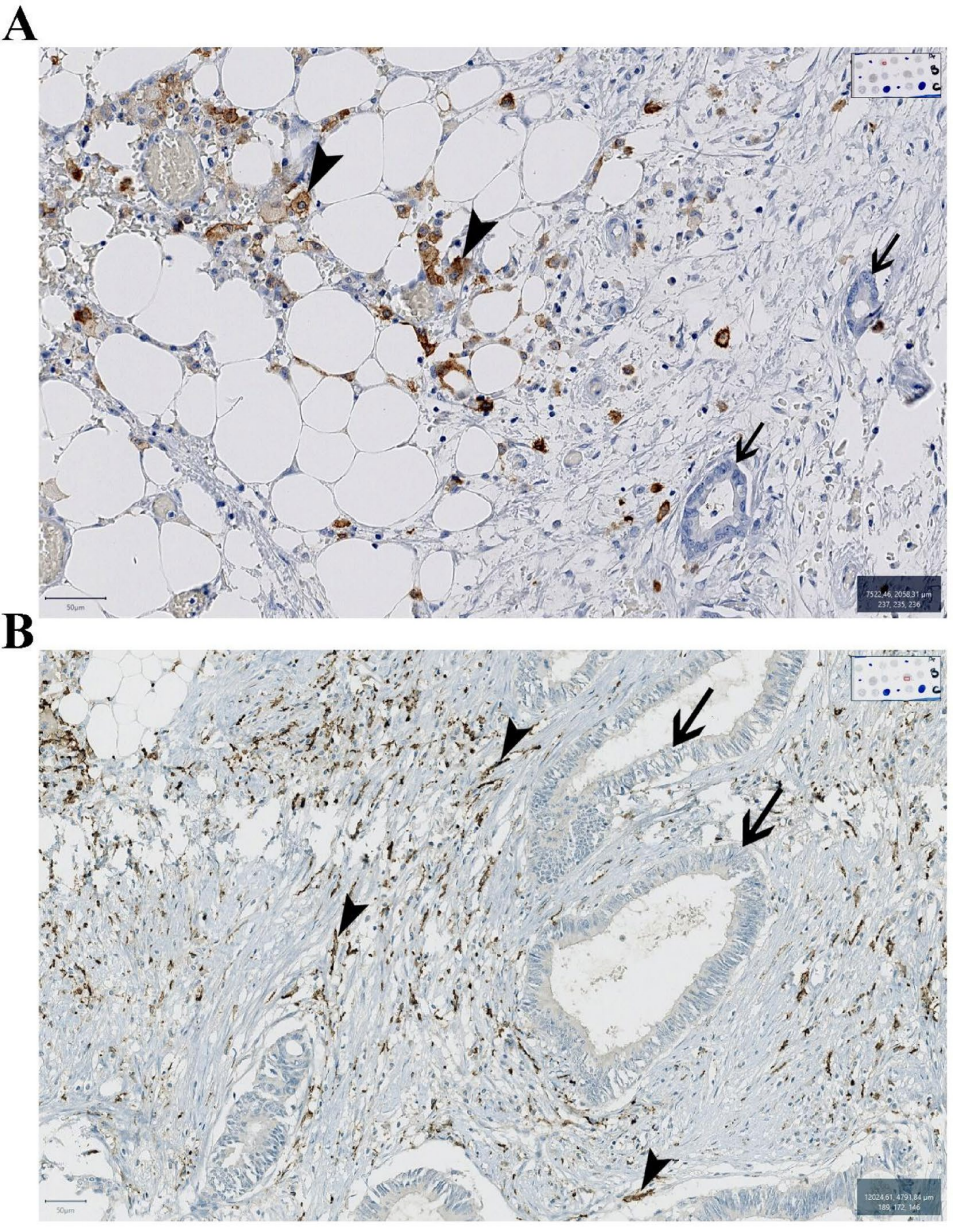
Macrophage Infiltration assessment. (**A**) CD163 staining showing round macrophages (arrowhead) and tumor cells (arrow). (**B**) CD163 staining showing spindle-shaped macrophages (arrowhead) and tumor cells (arrow).

Infiltration by spindle-shaped CD163 macrophages was linked to the CMS1 and CMS2/3 molecular subtypes, with a decreased presence of CMS4 (87.0% vs. 61.0%). A high immune score correlated with CD163 macrophage infiltration (both round and spindle-shaped) (Fig. 1).

### Survival analysis

**Fig. 2:**
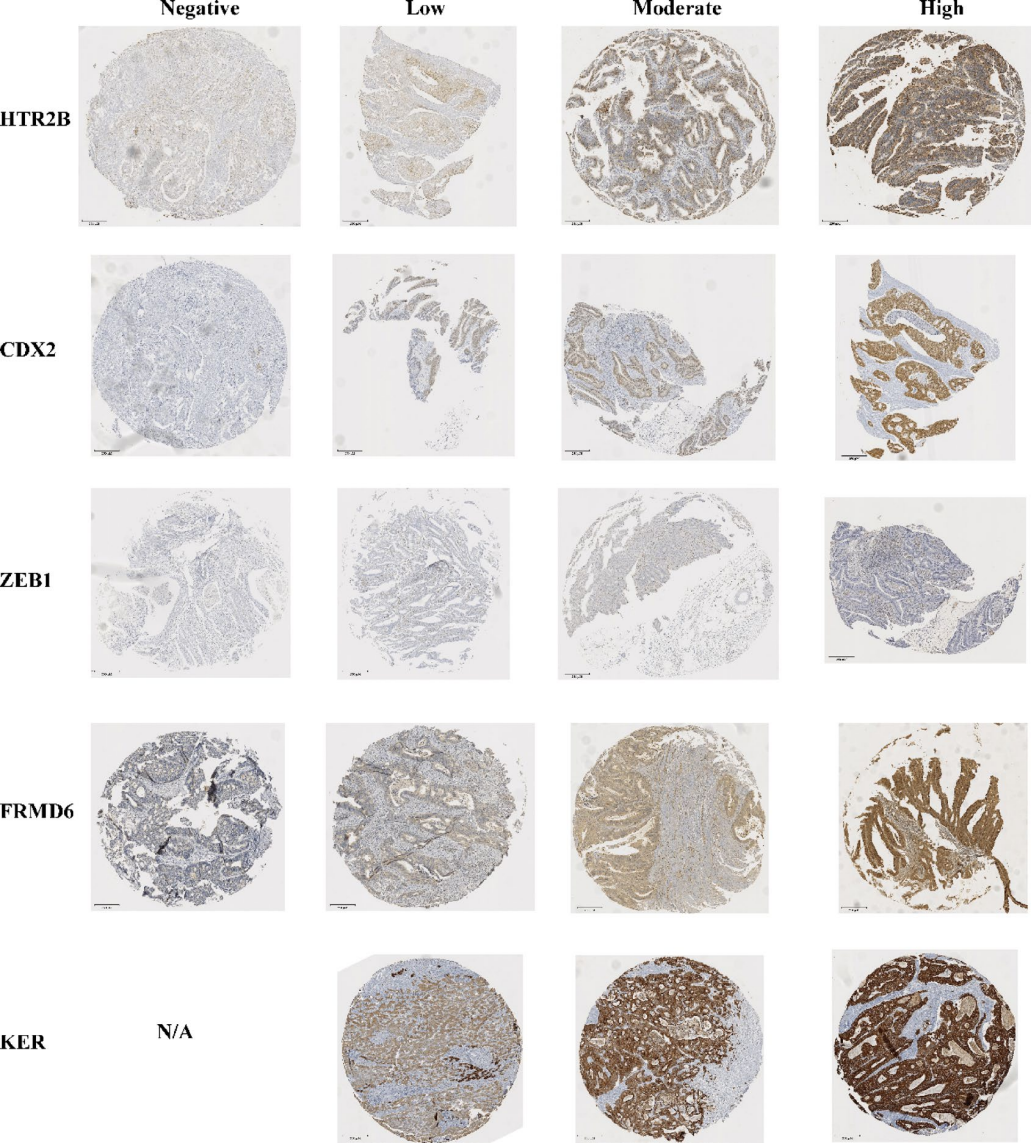
Tissue microarray IHC assessment of HTR2B, CDX2, ZEB1, FRMD6 and KER.

**Fig. 3:**
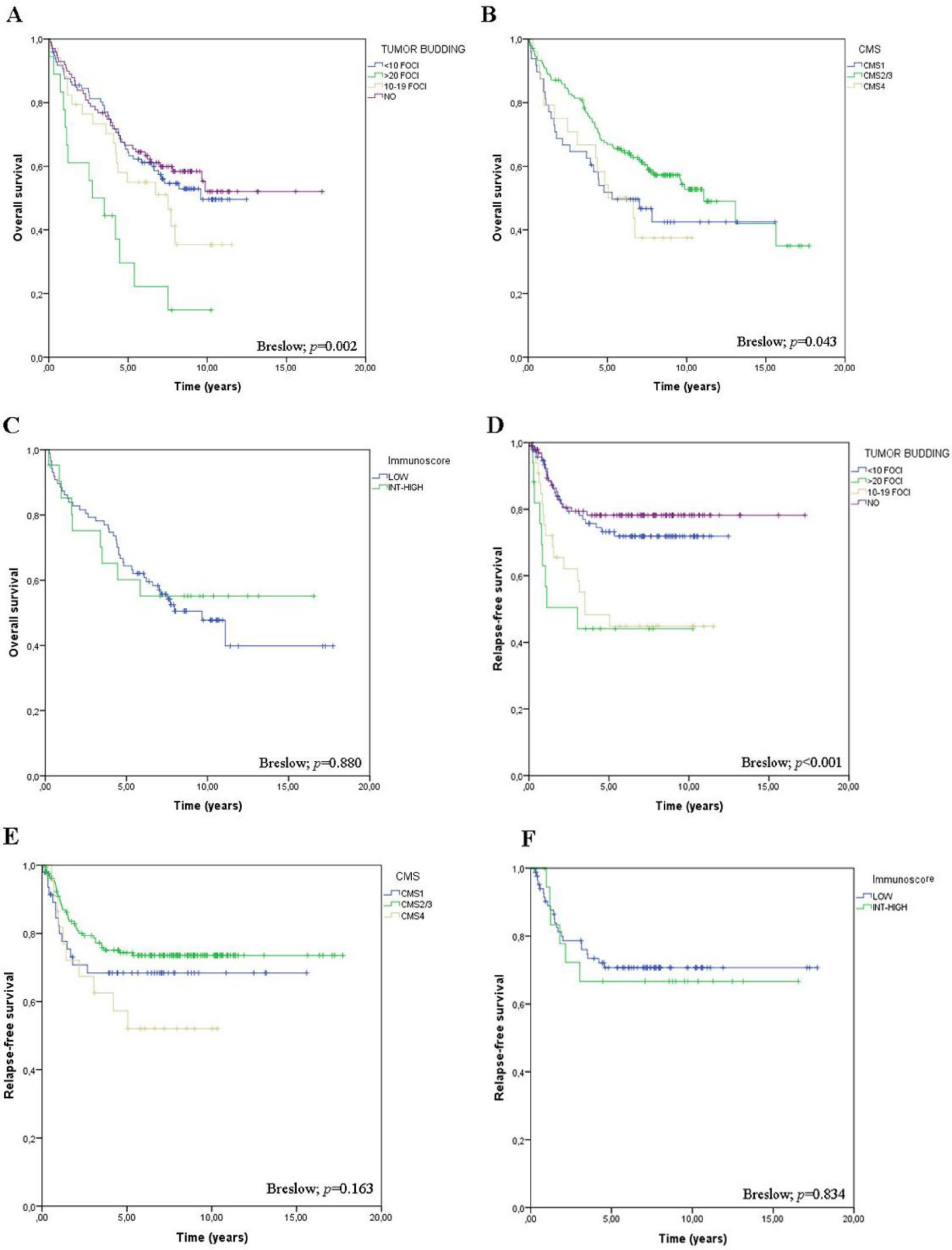
Kaplan–Meier survival curves illustrating the impact of different prognostic factors on overall and relapse-free survival. (**A**) Tumor budding, (**B**) consensus molecular subtype (CMS), and (**C**) Immunoscore in overall survival. (**D**) Tumor budding, (**E**) consensus molecular subtype (CMS), and (**F**) Immunoscore in relapse-free survival. Statistical significance was determined using the Breslow test.

As expected, in our cohort, survival analysis identified significant associations between overall and/or relapse-free survival and metastatic site at diagnosis; T, N, and M stages; histology; histologic grade; vascular, lymphatic, and perineural invasion; MSI status; *BRAF* mutations; infiltrative growth pattern; TB presence; CDX2 expression absence; and molecular subtypes (Figs. 2 and 3 and Tables 3, 4). For overall survival (OS), multivariate analysis revealed that sex (Hazard ratio, HR: 0.524, 95% CI: 0.340–0.806, adjusted *p* = 0.014), Eastern Cooperative Oncology Group performance-status scale (ECOG) (HR: 2.741, 95% CI: 1.994–3.767, adjusted *p* = 0.007) and histological grade (HR: 2.048, 95% CI: 1.410–2.975, adjusted *p* < 0.007) were significant predictors (Supplementary Fig. 1). These results highlight the critical prognostic role of functional status and tumour differentiation. Clinical stage showed no significant association with survival after adjustment (HR = 1.269; 95% CI, 0.964–1.669; adjusted *p* = 0.200); however, the observed effect direction was consistent with expectations. For relapse-free survival (RFS), clinical stage (HR, 2.336; 95% CI, 1.439–3.794; adjusted *P* = 0.009) emerged as a major predictor, reinforcing its role as a key determinant of prognosis (Supplementary Fig. 2). Although tumour budding and molecular subtypes did not show statistically significant associations with survival in this cohort, their directional trends were consistent with previously reported patterns and may be relevant in larger or stratified populations. These findings underscore the heterogeneity of CRC and multifactorial nature of CRC prognosis.

**Table 3:** Univariate survival analysis of clinical, pathological, and molecular features using the Breslow (generalized Wilcoxon) test.

	Overall survival	Relapse-free survival
	*p*-value	*p*-value
Sex	0.039	0.807
ECOG	< 0.001	0.061
T	0.001	< 0.001
N	< 0.001	< 0.001
M	< 0.001	< 0.001
Clinical stage	< 0.001	< 0.001
Sidedness	0.441	0.942
Polyps	0.972	0.211
CEA (presurgical)	0.053	0.005
Histological grade	0.028	< 0.001
MSI	0.005	0.719
CIMP	0.536	0.407
*BRAF*	0.014	0.921
*KRAS*	0.892	0.911
*NRAS*	0.116	0.260
CDX2 (positive/negative)	0.065	0.030
Tumor growth pattern	0.001	< 0.001
Tumor budding	0.003	< 0.001
Histology	0.015	0.566
Serrated adenocarcinoma	0.038	0.556
Vascular infiltration	< 0.001	< 0.001
Lymphatic infiltration	< 0.001	< 0.001
Perineural infiltration	0.001	< 0.001
Macrophages (CD163 spindle-shape)	0.861	0.665
Macrophages (CD163 round)	0.943	0.358
Macrophages (CD163 total)	0.754	0.846
Adjuvant treatment	0.002	0.585
Metastatic chemotherapy regimen	0.343	0.370
Metastatic location at diagnosis	0.361	0.419
Metastatic location at progression	0.240	0.002

**Table 4:** Multivariate survival analysis for overall and relapse-free survival.

	Overall survival				Relapse-free survival			
	HR	95%CI	*p*-value	FDR	HR	95%CI	*p*-value	FDR
Sex	0.524	0.340–0.806	0.003	0.014	-	-	-	
Clinical stage	1.269	0.964–1.669	0.089	0.200	2.369	1.461–3.841	< 0.001	0.009
ECOG	2.741	1.994–3.767	< 0.001	0.007	-	-	-	
Grade	2.048	1,410–2,975	< 0.001	0.007	1.936	1.093–3.428	0.023	0.104
MSI	1.069	.874–1.309	0.516	0.602	-	-	-	
BRAF	0.770	.386–1.538	0.459	0.584	-	-	-	
CEA	-	-	-		1.307	0.679–2.517	0.423	0.499
CDX	-	-	-		0.682	0.225–2.069	0.499	0.499
Growth pattern	1.794	1.004–3.207	0.048	0.134	1.683	0.746–3.797	0.210	0.378
Tumor budding	1.100	0.958–1.263	0.175	0.306	1.174	0.949–1.451	0.139	0.313
Histology	1.118	0.979–1.276	0.100	0.200	-	-	-	
Vascular infiltration	1.549	0.664–3.615	0.312	0.437	1.712	0.516–5.683	0.380	0.499
Lymphatic infiltration	1.355	0.783–2.346	0.277	0.431	2.034	0.891–4.646	0.092	0.276
Perineural infiltration	0.826	0.346–1.974	0.667	0.717	0.608	0.161–2.292	0.463	0.499
Adjuvant treatment	0.579	0.354–0.947	0.029	0.102	-	-	-	
CMS	0.911	0.550–1.509	0.717	0.717	-	-	-	

Hazard ratios (HR) are presented with their respective 95% confidence intervals (CI) to provide a comprehensive understanding of effect sizes and statistical significance. FDR-adjusted *p*-values using the Benjamini–Hochberg method.

## Discussion

Our study uniquely demonstrated a significant association between low immunoscore and high tumour budding in CMS2/3 subtypes of colorectal cancer, particularly in patients with serrated histology. This finding has not been extensively documented in the literature and provides new insights into the complex interplay between molecular subtypes, immune responses, and tumour behaviour. These findings could guide clinical trials focusing on tumour characteristics and immune microenvironment. Conventional and serrated tumours are associated with lower immunoscore. The molecular subtype distribution of our cohort differed from that of Guinney et al. (2015)^10^, who showed more CMS1 (20.45%) and fewer CMS4 (10.2%) subtypes, likely due to patient selection based on stage and histology (mostly localised disease at 81.2%). Because differentiation between CMS2 and CMS3 by immunohistochemistry is not yet possible, we combined them into one group: CMS2/3. Epithelial subtypes (CMS2/3) were the most prevalent (69.4%) and were often associated with epithelial characteristics and a better prognosis. CMS1, characterised by immune activation and high microsatellite instability (MSI-H), forms a small subset but is associated with favourable immunotherapy responses and poor prognosis in advanced stages owing to immune evasion^29^. In addition to the findings presented, CMS1 tumours, characterised by high immune infiltration and microsatellite instability (MSI-H), are known to be particularly immunogenic and responsive to immune checkpoint inhibitors. This aligns with studies highlighting the efficacy of immunotherapy in MSI-H colorectal cancers owing to their high mutational burden and neoantigen presentation. Given the strong immune activity associated with CMS1, our study reinforces the potential value of immunotherapy as a promising treatment strategy for this subgroup, especially in the advanced stages, and underscores the need for further research to optimise its use in clinical practice. CMS4, known for its mesenchymal features and aggressive behaviour, has the poorest prognosis, increased metastasis, and therapy resistance^30^. Our dataset confirms these findings in terms of survival and CMS. Our findings that the CMS1 and CMS4 subtypes were associated with poorer overall survival align with recent transcriptomic analyses. For example, Yang et al. (2025)^31^ reported reduced OS in both CMS1 and CMS4 using large-scale gene expression data from patients with colorectal cancer. Although their classification was RNA-based and our IHC-based, parallel trends reinforce the notion that immune-rich (CMS1) and mesenchymal (CMS4) tumours may exhibit aggressive clinical behaviour, albeit via distinct pathways such as immune escape and stromal remodelling, respectively.

Adjuvant treatment was administered to 40.8% of the patients, predominantly using the CAPOX regimen, which combines capecitabine and oxaliplatin^32^. The efficacy of the CAPOX regimen in stage III colon cancer, as highlighted by Twelves et al.^32^, demonstrates a balance between effectiveness and manageable toxicity. Among the patients with localised disease, 40 experienced recurrences, predominantly in the CMS2/3 (57.5%) group, followed by the CMS1 (20.0%) and CMS4 (10.0%) groups. This recurrence pattern suggests that CMS2/3, despite being more common, carries a substantial risk of recurrence, necessitating closer surveillance and possibly more aggressive adjuvant therapy, even though CMS2 has the best OS after relapse^10^. We found that CMS2/3 had better survival after adjuvant chemotherapy than CMS1 and CMS4 did. Our findings indicate that the CMS2/3 subtypes have better survival outcomes after adjuvant chemotherapy than CMS1 and CMS4, which is consistent with previous studies (e.g. Dienstmann et al. 2017; Guinney et al. 2015)^10,30^ that have highlighted the responsiveness of epithelial subtypes to systemic therapies. However, other investigations have shown survival benefits in CMS1, CMS2, and CMS4, but no clear advantage for CMS3^33^. This discrepancy could arise from variations in the study design, sample size, treatment regimens employed in the adjuvant setting, or differences in outcome measures.

The CMS2 and CMS3 subtypes are both epithelial, but differ in their key molecular characteristics. CMS2 is driven by WNT and MYC pathway activation and is often associated with high chromosomal instability (CIN), while CMS3 exhibits metabolic reprogramming and frequent *KRAS/PI3K* mutations. These distinctions may influence the differential benefits of chemotherapy. Studies focusing on metastatic CRC often underscore chemotherapy sensitivity in CMS2 and CMS3; however, evidence on non-metastatic CRC remains limited and inconsistent^34–36^. Future research, particularly in prospective, larger cohorts, is needed to clarify these findings and to further explore the mechanisms underlying subtype-specific responses to treatment.

Immunoscore analysis indicated that 80.6% of patients had low immunoscore, whereas 19.4% had intermediate-to-high scores. Immunoscore is a significant prognostic marker, with higher scores suggesting a better immune response and improved outcomes^13,37,38^. While the original Immunoscore divides patients into five categories (I0–I4), we opted to simplify the classification into a binary variable (low: I0–I1, intermediate-to-high: I2–I4). This decision aligns with the validated approach used by Pagès et al.^13^, which has demonstrated the prognostic utility of the binary classification in colorectal cancer. Moreover, our analysis confirmed that the results were consistent whether the Immunoscore was treated as a binary variable or across the five original categories. This consistency suggests that the binary classification maintains the prognostic value while providing a more practical framework for clinical application. Low immunoscore are commonly associated with conventional and serrated adenocarcinomas, with serrated adenocarcinomas showing lower immune responses and less lymphocyte infiltration^39^ despite being classified under the CMS4 subtype^40^. Histological and molecular classifications of CRC diverge, and another subtype of serrated adenocarcinoma ("classical serrated CRC") has been proposed to belong to the CMS1 subtype^40,41^. In our cohort, MSI-H status was linked to intermediate-high immunoscore, aligning with Galon et al. (2006)^42^ that MSI-H tumors are more immunogenic and elicit a stronger immune response. International validation of the immunoscore demonstrated its robust predictive power for survival in patients with colon cancer, outperforming traditional clinicopathological factors. Incorporating immunoscore into clinical practice can enhance risk stratification and therapeutic decision making^13^.

The metastatic patterns at diagnosis varied by molecular subtype: CMS1 had peritoneal metastases (50.0%), CMS2/3 had hepatic metastases (61.5%), and CMS4 had peritoneal (72.7%) and hepatic (30.0%) metastases. These results suggest that molecular subtyping provides insights into metastatic pathways, thus informing tailored surveillance strategies^10^. The progression/relapse patterns also depended on the subtype: CMS1 with peritoneal (42.9%) and nodal (28.6%) progression, CMS2/3 with hepatic (40.0%) and pulmonary (32.5%) progression, and CMS4 with peritoneal (50.0%) and hepatic (30.0%) progression.

Studies have confirmed that molecular subtypes can predict metastatic patterns, treatment responses, and prognosis^10,43^. For instance, patients with CMS1 respond better to immunotherapy owing to their high mutational burden and immunogenic neoantigens^44^. Conversely, CMS4 patients exhibit lower responses to standard chemotherapy and immunotherapy because of their pro-migratory and pro-angiogenic attributes^45^.

Building on insights from immunoscore analysis, we examined the patterns of tumor budding and their clinical implications. Tumor budding (TB) was notably present in serrated (82.5%), comedo-cribriform (71.4%), and conventional (65.4%) histology. Defined as single cells or small clusters of up to four cells at the invasive front of the carcinoma, TB is a recognized adverse prognostic factor for CRC^46^. Our findings corroborate that TB is significantly associated with a high recurrence rates^47^. The cutoff values for tumor budding (< 10, 10–19, ≥ 20) were selected based on their clinical relevance and ability to stratify patients effectively into prognostic groups. These thresholds align with previously utilized ranges in studies like Ueno et al.^48^, which similarly categorized TB to evaluate its prognostic significance in colorectal cancer. While these specific values are not universally standardized, they were adapted to suit the distribution and characteristics of our cohort. Among the patients who experienced recurrence, 70.0% had TB-positive tumors, underscoring role of TB in disease progression and its potential as a therapeutic target^47^. TB is associated with more aggressive tumor characteristics and poorer overall and disease-free survival^48^. TB assessment aids in clinical decision making by identifying patients who may benefit from more intensive treatment^46^.

High TB (> = 20 foci) was correlated with hepatic progression (66.7%), 10–19 foci with both hepatic and peritoneal progression (29.4%), and < 10 foci with hepatic (40.0%), pulmonary (36.0%), and peritoneal (24.0%) progression. The absence of TB was linked to peritoneal (45.0%) and pulmonary (20.0%) disease progressions. These findings highlight the aggressive nature of tumors with high TB and their tendency for liver metastases, corroborating studies identifying TB as a marker of aggressive behaviour and poor prognosis^48^. TB evaluation is particularly useful in early stage CRC, indicating a higher risk of progression and recurrence, thus warranting a more aggressive therapeutic approach^47^. Additionally, TB is associated with other aggressive markers such as vascular and lymphatic invasion, reinforcing its prognostic value^48^.

The infiltration of CD163+ macrophages, indicative of an immunosuppressive M2 phenotype, varied among colorectal cancer (CRC) subtypes, being most prevalent in CMS1 and CMS2/3 (87.0% vs. 61.0%), less prevalent in CMS4, and associated with an intermediate-to-high immunoscore. M2 macrophages promote angiogenesis, immunosuppression, and tumor microenvironment remodelling, thereby affecting disease outcomes^49,50^. However, they also limit metastasis by influencing vascular maturity and normalization^51^. The modulation of macrophage polarization is a promising therapeutic strategy^50^.

While previous studies have separately established the prognostic value of immunoscore and tumor budding, our results revealed a previously unexplored relationship between these factors within specific molecular subtypes. This adds a new dimension to our understanding of CRC heterogeneity and potential treatment strategies. These results directly address the gaps identified in the current research by demonstrating the association between a low immunoscore and high tumor budding in CMS2/3 subtypes, suggesting that these tumors may have developed mechanisms to evade immune surveillance while maintaining aggressive behaviour. TB is an emerging prognostic biomarker in colorectal cancer (CRC) that currently influences decision-making in stage I and II CRC. Studies, such as the post-hoc analysis of the IDEA-France trial, have demonstrated that intermediate and high TB are significantly associated with poorer disease-free survival (DFS) and overall survival (OS) in stage III CRC patients. Furthermore, the relationship between TB and Immunoscore is an underexplored area of research. Recent evidence^52^ highlighted that combining Immunoscore with TB improves prognostic accuracy compared to traditional factors such as N-stage, T-stage, and MSI. This combination also had the highest relative contribution to DFS in early-stage CRC. However, given the limited existing evidence, our findings contribute novel insights into this field by demonstrating a significant association between low Immunoscore and high TB in CMS2/3 subtypes of CRC, particularly in serrated histology. This association suggests that these tumors may evade immune surveillance while exhibiting aggressive behaviour, as reflected in higher recurrence rates. These findings reinforce the need for further studies to validate and expand on these observations, particularly in prospective and larger-scale cohorts, as they provide important implications for refining risk stratification and tailoring therapeutic approaches in CMS2/3 patients.

Although our study provides valuable insights, it is limited by its retrospective nature and small sample size. The potential impact of prior treatments on immune response, tumor behaviour, and survival outcomes must be considered when interpreting these results. In our cohort, 40.8% of patients with localized disease received adjuvant treatment, predominantly CAPOX. This regimen, combining capecitabine and oxaliplatin, has demonstrated efficacy in improving survival in stage III colorectal cancer patients, but it may also alter the tumor microenvironment and immune response. Beyond cytotoxic efficacy, CAPOX may modulate local immunity. Oxaliplatin induces immunogenic cell death characterised by calreticulin exposure and HMGB1 release, enhancing antigen cross-presentation^53^, whereas capecitabine can transiently reduce myeloid-derived suppressor cells^5^. Conversely, fluoropyrimidine-related lymphopenia may blunt the lymphocyte-mediated responses. Such counteracting effects could partially account for the heterogeneous immunoscore values recorded after adjuvant therapy. Such changes could affect variables, such as tumour budding and immune infiltration, potentially introducing variability into our findings. Despite these complexities, the inclusion of patients with diverse treatment backgrounds enriches the dataset, allowing us to examine the prognostic value of variables such as immunoscore and consensus molecular subtypes across a real-world population. Future prospective studies with larger cohorts are needed to validate these findings and to explore their potential clinical applications, such as tailoring immunotherapy approaches based on molecular subtypes and immunoscore.

Canonical WNT/β-catenin and MYC signalling—hallmarks of epithelial CMS2 and, to a lesser degree, CMS3—provide a plausible explanation for the low immune/high budding phenotype we observed. Constitutive β-catenin activity suppresses CCL4 and hinders the recruitment of dendritic cells and cytotoxic T lymphocytes, thereby creating an immune-excluded micro-environment^54^. In parallel, WNT-β-catenin and MYC drive epithelial–mesenchymal transition via ZEB1 upregulation, histologically manifesting as high-grade budding^45^. These convergent pathways reinforce the rationale for trials that combine WNT/MYC inhibitors with immunotherapy in CMS2/3 tumours. Our immunohistochemistry mini-classifier could not separate CMS2 from CMS3; both were aggregated into a single epithelial group. Although this approach shows substantial concordance with transcriptome-based CMS calling (κ ≈ 0.75)^7^, residual misclassification may have diluted subtype-specific signals, particularly those unique to the metabolic CMS3 subgroup.

In conclusion, this study highlights the intricate interplay between molecular subtypes, immune responses, and tumour behaviours in colorectal cancer (CRC), emphasising the need for a transformative approach to clinical strategies and research. By demonstrating that the CMS2/3 subtypes, especially serrated tumours, exhibit low immune responses and high tumour budding linked to hepatic metastasis, our findings underline the importance of tailoring clinical interventions and surveillance. Additionally, while acknowledging the limitations of this study, future research should explore the integration of genetic, immunological, and morphological data to refine personalised treatment pathways and enhance patient outcomes. These results not only enhance our understanding of CRC heterogeneity, but also provide actionable advocacy for advancing precision medicine in this field.

## Methods

### Study design and patient cohort

This retrospective observational cohort study was conducted using an existing database, not previously published, from the pathological anatomy department of our hospital, in which 255 patients were selected according to available metadata and histological subtypes, mainly in localised stages, although metastatic patients were included. A pathologist selected samples from the resected specimens using TMA cores. The selected patients were under the care of the Medical Oncology Service at the Hospital General Universitario Santa Lucía, Cartagena. Demographic, histopathological, and clinical variables were also analysed. Molecular classification, immunoscore, and macrophage infiltration were determined by immunohistochemistry.

### Tissue microarray construction and validation

Two regions per case, selected by the pathologist, were cored to construct the TMAs using a manual tissue microarrayer (MTA-1, Beecher Instruments, Wisconsin, USA). Two cores (1.5-mm diameter, total sampled area ≈ 3.53 mm^2^) were taken per case at the invasion front, selected by an experienced gastrointestinal pathologist (J.G-S.) after reviewing the corresponding whole-slide H&E-stained sections. To verify representativeness, we re-evaluated immunoscore and budding on whole sections in a random 15.0% subset (n = 38), and concordance between the whole section and TMA was excellent (κ = 0.83 for immunoscore, κ = 0.79 for tumour budding).

### CMS assessment

Immunohistochemical staining was performed on TMAs as previously described by Conesa-Zamora et al.^55^ using automated Window BENCHMARK equipment, with staining for HTR2B (anti-HTR2B (26408-1-AP), 1:100, Fisher Scientific, Madrid, Spain), FRMD6 (anti-FRMD6, 1:500, Sigma, Madrid, Spain), ZEB1 (anti-ZEB1, 1:500, Sigma, Madrid, Spain), pancytokeratin, CDX-2 (EPR2764Y), CD163 (MRQ-26), CD14 (EPR3653), CD8 (SP57), and CD3 (2GV6)—were supplied by Roche, headquartered in Madrid, Spain. The molecular subtype was then determined using the Online Immunohistochemistry (IHC)-Mini Classifier, available at: https://crcclassifier.shinyapps.io/appTesting/^7^. The IHC mini-classifier has previously shown substantial agreement with gene-expression-based CMS calls (κ = 0.76; 84.0% raw concordance) in an independent multi-centre series^7^. Although we adopted an identical antibody panel and scoring rules, no local transcriptomic validation was feasible and potential misclassification could not be excluded. The classification thresholds and decision rules for allocating tumours to CMS1, CMS2/3, or CMS4 were predefined within the online classifier algorithm and applied without modification. These are based on published validation datasets integrating marker expression intensity, localisation, and morphological features, as described by Anne Trinh^56^ and validated by Hoorn et al.^7^.

### Immunoscore calculation

Immunohistochemical evaluation of CD3+ and CD8+ stained immune cells was performed on TMAs, as described by Conesa-Zamora et al.^55^. CD3⁺ and CD8⁺ T-cell densities were assessed manually by two independent pathologists. For each case, we selected the hotspot at the invasive margin and counted the positive cells in four non-overlapping high-power fields at ×40 magnification. Densities (cells mm⁻^2^) were averaged per compartment and converted to a five-tier immunoscore (I0–I4) according to the thresholds of Pagès et al.^13^. Inter-observer reproducibility, calculated for 25 randomly chosen tumours, showed a strong correlation (Pearson r = 0.89). The Immunoscore was calculated following the method described by Jiang et al.^57^, which divides cases into five categories (I0–I4) based on the density of CD3+ and CD8+ T cells in the tumour centre and invasive margin. For the purposes of this study, these five categories were further simplified into two groups: low immunoscore (I0–I1) and intermediate-to-high immunoscore (I2–I4). This binary categorisation was chosen to enhance clinical interpretability and was consistent with the approach validated in other studies, such as Pagès et al.^13^. Moreover, our analysis confirmed that the results were consistent regardless of whether Immunoscore was treated as a binary variable or across the five original categories, demonstrating the robustness of this approach (Table 2 and Supplementary Table 2).

### Tumour budding

Tumour budding was quantified on duplicate 1.5-mm tissue cores stained with H&E. Budding foci were counted across the full core area (total ≈3.5 mm^2^ per case) and categorised into three groups: < 10, 10–19, and ≥ 20 buds, similar to previous literature^48^. These thresholds differ from the ITBCC 2016 classification, which is based on a single 0.785 mm^2^ hotspot and are not directly comparable^22^.

### Macrophage assessment

Immunohistochemical evaluation of CD163+ stained immune cells was performed on TMAs as described by Conesa-Zamora et al.^55^. M2 macrophages were then classified according to their shape into spindle-cell and round-cell macrophages.

### Assessment of CpG Island methylation phenotype (CIMP)

We used methylation-specific multiplex ligation-dependent probe amplification (MS-MLPA) with the CIMP-specific SALSA probemix ME042-C1 (MRC-Holland, Amsterdam) as previously described in Turpín-Sevilla et al.^58^. For DNA input, 100 ng of tumour DNA per case was denatured in 5 µL Tris–EDTA buffer (TE), and fragment analysis was performed on an ABI 3130 capillary sequencer. Normal reference DNA: pooled normal colonic mucosa. Data processing: Coffalyser.net™ (default settings), inter-sample normalisation against multiple runs of the reference DNA, and intra-sample normalisation to the reference probes of the kit. The peak-height ratios (digested: undigested) were used to score each probe. Genes showing partial methylation were called “methylated” following the manufacturer’s instructions. Classification criteria: We applied the following standard scheme. Weisenberger et al.^8^. 5-gene panel (CIMP-W) — CIMP-High if > 3 of 5 loci (*CACNA1G*, *IGF2*, *NEUROG1*, *RUNX3*, *SOCS1*) methylated, CIMP-Negative if ≤ 3.

### Statistical analysis

The statistical rationale of this study was designed to provide a detailed and comparative description of numerical and categorical variables as well as to evaluate survival and associated risk factors. Statistical analyses were performed using IBM SPSS Statistics version 21 (IBM Corp., Armonk, NY). Numerical variables were represented by the median and range, and categorical variables were described as counts and relative frequencies in each category. Comparisons of categorical variables between two or more groups were performed using contingency tables and chi-square tests. Survival curves were estimated using the Kaplan–Meier method and univariate survival analyses were performed using the Breslow (generalized Wilcoxon) test. To complement these analyses and provide hazard ratios (HR) with 95% confidence intervals (CI), univariate Cox proportional hazards models were additionally generated. The corresponding HRs, CIs, and *p*-values are reported in Supplementary Table 3. Multivariate analyses were conducted using Cox proportional hazards regression models, which were chosen for their ability to assess the simultaneous impact of multiple variables on survival outcomes while accounting for confounding factors. Confounder selection for the multivariate models was guided by both statistical significance in univariate analysis (*p* < 0.05) and clinical relevance. The variables included were clinical stage, ECOG performance status, histological grade, vascular and lymphatic invasion, tumour growth pattern, tumour budding, immunoscore, and consensus molecular subtypes (CMS), all of which have established prognostic implications in colorectal cancer. *P*-values from the multivariate Cox models were adjusted for multiple testing using the false discovery rate (FDR) method according to Benjamini–Hochberg. Variables with FDR-adjusted *p*-values < 0.05 were considered significant^59^. Descriptive statistical analyses were performed to examine the association between the molecular subtypes (CMS) and metastatic patterns at diagnosis. This included the use of contingency tables and chi-square tests to identify significant relationships. These methods allowed us to evaluate whether trends in metastatic sites, such as hepatic or peritoneal metastases, differed among CMS1, CMS2/3, and CMS4. These associations were descriptive and did not include hazard ratios (HR) or relative risk (RR).

## Limitations

This study has several limitations. First, though desirable for the sake of representativity, the selection of patients based on histological subtypes, rather than random sampling, may have introduced selection bias and limits the representativeness of the cohort. This retrospective enrichment, particularly for serrated and mucinous phenotypes, could have shifted the immune micro-environment landscape. In addition, patients were excluded if insufficient tumour tissue was available for immunohistochemical analysis, which reduced the sample size for some variables and limited the power of certain subgroup analyses. Missing data were handled through case-wise exclusion, with sample sizes explicitly reported to ensure transparency.

Second, the classification of molecular subtypes relied on an immunohistochemical mini-classifier rather than transcriptome-based profiling. Although this method has shown substantial concordance with gene expression-based CMS calls (κ ≈ 0.75), it cannot reliably separate CMS2 from CMS3 or identify the NOS (mixed) category. Residual misclassification may therefore have affected subtype-specific associations. Moreover, there is limited and inconsistent evidence regarding CMS3 in early-stage CRC, which could partly explain discrepancies with prior studies.

Third, all immune and tumour-budding evaluations were performed on triplicate 1.5-mm tissue micro-array cores, rather than on whole sections. Although we observed substantial concordance with whole-slide estimates in a validation subset (n = 38), core-based sampling cannot fully capture spatial heterogeneity, particularly in immune-excluded CMS2/3 tumours. Additionally, tumour budding was quantified across the entire core area and categorised as < 10, 10–19, or ≥ 20 foci. These thresholds, while informed by previous studies, differ from the ITBCC 2016 standards based on a single 0.785 mm^2^ hotspot, and are therefore not directly comparable.

Furthermore, we did not stratify our analysis of metastatic patterns by the anatomical location of the primary tumour. Rectal cancers represented 18.8% of our cohort and may follow different dissemination routes compared to colonic tumours, such as a higher likelihood of lung rather than liver metastases. This anatomical variability could act as a confounder in the associations observed between tumour budding grade and metastatic site. Future studies should address this by incorporating tumour location into multivariate analyses of metastatic tropism.

Finally, to improve interpretability, we dichotomised the original five-tier Immunoscore and conducted multiple exploratory comparisons. Sensitivity analyses using the full I0–I4 scale yielded consistent results, and we applied false-discovery-rate correction to reduce the risk of type I error. Nonetheless, the small size of some subgroups—such as CMS4 or relapsed patients with evaluable immune scores—may have limited statistical power and increased the potential for type II error.

Despite these limitations, our study provides important insights into the interplay between molecular subtypes, immune responses and tumour behaviour in CRC. Future prospective studies with larger, more diverse cohorts and transcriptomic validation will be key to confirming and extending these findings.

## Electronic supplementary material

Below is the link to the electronic supplementary material.

Supplementary Material 1

## Data Availability

The data that support the findings of this study are not openly available due to reasons of sensitivity and are available from the corresponding author upon reasonable request. Data are located in controlled access data storage at Hospital General Universitario Santa Lucía.

## Abbreviations

CAPOX: Capecitabine plus oxaliplatin adjuvant chemotherapy regimen
CEA: Carcino-embryonic antigen
CIMP: CpG-island methylator phenotype
CI: Confidence interval
DFS: Disease-free survival
ECOG: Eastern Cooperative Oncology Group performance-status scale
EMT: Epithelial–mesenchymal transition
HR: Hazard ratio
IHC: Immunohistochemistry
IFN-γ: Interferon-gamma
IL1-β: Interleukin-1 beta
ITBCC: International Tumour Budding Consensus Conference
LPS: Lipopolysaccharide
MS-MLPA: Methylation-specific multiplex ligation-dependent probe amplification
MSS: Microsatellite stable
OS: Overall survival
RFS: Relapse-free survival
TE: Tris–EDTA buffer
TMA: Tissue micro-array
TNF: Tumour necrosis factor
TNM: Tumour-node-metastasis staging system
