# Supplementary material for: Clinical and immunopathological evaluation and its comparison with IHC consensus molecular subtypes of colorectal cancer
Source: Sci Rep. 2025 Jul 1;15:21626. doi: 10.1038/s41598-025-04962-w (PMC12215256; doi:10.1038/s41598-025-04962-w)
Supplement: Supplementary file 1 — Supplementary Material 1 [file 41598_2025_4962_MOESM1_ESM.pdf]

# Clinical and immunopathological evaluation and its comparison with IHC consensus molecular subtypes of colorectal cancer

Eduardo Feliciangeli[1,2,3,*], Ana Albaladejo-González[3,4], José García-Rodríguez[4], Antonio Lázaro-Sánchez[2,6], Rosanna Borg[5], Paola Pimentel-Cáceres[1,2], Diego Soriano-Polo[1], Edith Rodríguez-Braun[1,2], José Balsalobre-Yago[1,2,3], María José Martínez-Ortiz[1,2,3], Sofía Wikström-Fernández[1,2,3], Andrés Murillo-Herrera[1], Teresa García-García[1,3], José García-Solano[2,3,4], Pablo Conesa-Zamora[2,3,4,*] and Ginés Luengo-Gil[2,3,4,*]

[1] Medical Oncology Department, Hospital General Universitario Santa Lucía, Cartagena, Spain.
[2] Group of Molecular Pathology and Pharmacogenetics, Instituto Murciano de Investigación Biosanitaria (IMIB), Hospital Universitario Santa Lucía, Cartagena, Spain.
[3] Health Sciences Faculty, Universidad Católica de Murcia (UCAM), Guadalupe, Spain.
[4] Laboratory Medicine and Pathology Department, Hospital General Universitario Santa Lucía, Cartagena, Spain.
[5] Van Hall Larenstein University of Applied Sciences, Agora 1, P.O. Box 1528, 8901 BV Leeuwarden, Netherlands.
[6] Medical Oncology Department, Hospital General Universitario Morales Meseguer, Murcia, Spain.
* Correspondence: EFM: edufeliciangeli@gmail.com; PCZ: pconesa@ucam.edu; GLG: gluengo@ucam.edu; Tel.: (+34 968128602 ext: 951439)

## SUPPLEMENTARY MATERIAL

**Supplementary Table S1**. Distribution of Immunoscore categories by CMS subtype.

| Immunoscore Category | CMS1 (n=18) | CMS2/3 (n=79) | CMS4 (n=9) | Total (n=106) |
|---|---|---|---|---|
| Low (I0–I1) | 10 | 67 | 8 | 85 |
| Intermediate–High (I2–I4) | 8 | 12 | 1 | 21 |

Low Immunoscore includes categories I0–I1; Intermediate–High Immunoscore includes categories I2–I4. CMS subtype assignment was based on immunohistochemical classifier results.

**Supplementary Table 2.** Association of clinical and pathological variables with immunoscore (I0-I4).

| | Immunoscore | |
|---|---|---|
| | n | *p*-value |
| **Age at diagnosis*** <br> **Median and interquartile range: 68.8 years [IQR 61–77]** | I0: 59<br>I1: 28<br>I2: 18<br>I3: 2<br>I4: 1 | 0.506 |
| **Sex** | | 0.512 |
| Male | I0: 33<br>I1: 19<br>I2: 10<br>I3: 2<br>I4: 1 | |
| Female | I0: 26<br>I1: 9<br>I2: 8<br>I3: 0<br>I4: 0 | |

| | | |
|---|---|---|
| **ECOG** | | |
| 1 | I0: 42<br>I1: 19<br>I2: 11<br>I3: 2<br>I4: 0 | |
| 2 | I0: 10<br>I1: 7<br>I2: 4<br>I3: 0<br>I4: 1 | |
| 3 | I0: 6<br>I1: 2<br>I2: 3<br>I3: 0<br>I4: 0 | 0.823 |
| 4 | I0: 1<br>I1: 0<br>I2: 0<br>I3: 0<br>I4: 0 | |
| **Clinical stage** | | |
| I | I0: 7<br>I1: 6<br>I2: 0<br>I3: 0<br>I4: 1 | |
| II | I0: 23<br>I1: 6<br>I2: 6<br>I3: 0<br>I4: 0 | 0.137 |
| III | I0: 22<br>I1: 11<br>I2: 8<br>I3: 2<br>I4: 0 | |
| IV | I0: 6<br>I1: 5<br>I2: 4<br>I3: 0<br>I4: 0 | |
| **Sidedness** | | |
| Left | I0: 27<br>I1: 20<br>I2: 7<br>I3: 1<br>I4: 1 | 0.120 |
| Right | I0: 32<br>I1: 8<br>I2: 11<br>I3: 1<br>I4: 0 | |
| **Colon or rectum** | I0: 48 | 0.532 |

| | | |
|---|---|---|
| Colon | I1: 25<br>I2: 16<br>I3: 1<br>I4: 1 | |
| Rectum | I0: 11<br>I1: 3<br>I2: 2<br>I3: 1<br>I4: 0 | |
| **Polyps** | | |
| Yes | I0: 36<br>I1: 16<br>I2: 10<br>I3: 1<br>I4: 1 | 0.912 |
| No | I0: 23<br>I1: 12<br>I2: 8<br>I3: 1<br>I4: 0 | |
| **Carcino-embryonic antigen, CEA (presurgical)** | | |
| =<5 | I0: 29<br>I1: 16<br>I2: 10<br>I3: 1<br>I4: 1 | 0.931 |
| >5 | I0: 11<br>I1: 7<br>I2: 4<br>I3: 0<br>I4: 0 | |
| **Histological grade** | | |
| 1 | I0: 14<br>I1: 11<br>I2: 5<br>I3: 0<br>I4: 0 | |
| 2 | I0: 38<br>I1: 16<br>I2: 12<br>I3: 2<br>I4: 1 | 0.780 |
| 3 | I0: 6<br>I1: 1<br>I2: 1<br>I3: 0<br>I4: 0 | |
| **MSI** | | |
| Yes | I0: 6<br>I1: 2<br>I2: 6<br>I3: 1<br>I4: 1 | 0.006 |
| No | I0: 51<br>I1: 26<br>I2: 12<br>I3: 1 | |

| | | |
|---|---|---|
| | I4: 0 | |
| **CIMP** | | |
| No | I0: 10<br>I1: 2<br>I2: 3<br>I3: 0<br>I4: 0 | |
| Low | I0: 4<br>I1: 5<br>I2: 1<br>I3: 0<br>I4: 0 | 0.368 |
| **High** | I0: 3<br>I1: 1<br>I2: 1<br>I3: 0<br>I4: 0 | |
| ***BRAF*** | | |
| Native | I0: 53<br>I1: 27<br>I2: 16<br>I3: 2<br>I4: 1 | 0.859 |
| Mutated | I0: 5<br>I1: 1<br>I2: 2<br>I3: 0<br>I4: 0 | |
| ***KRAS*** | | |
| Native | I0: 25<br>I1: 11<br>I2: 10<br>I3: 1<br>I4: 1 | 0.643 |
| Mutated | I0: 33<br>I1: 17<br>I2: 8<br>I3: 1<br>I4: 0 | |
| ***NRAS*** | | |
| Native | I0: 52<br>I1: 24<br>I2: 15<br>I3: 2<br>I4: 0 | 0.109 |
| Mutated | I0: 6<br>I1: 4<br>I2: 3<br>I3: 0<br>I4: 1 | |
| **CDX2** | | |
| Positive | I0: 51<br>I1: 24<br>I2: 15<br>I3: 1<br>I4: 0 | 0.017 |
| Negative | I0: 5<br>I1: 3<br>I2: 1<br>I3: 1 | |

| | | |
|---|---|---|
| | I4: 1 | |
| **Tumor growth pattern** | | |
| Infiltrative | I0: 21<br>I1: 9<br>I2: 4<br>I3: 0<br>I4: 0 | 0.610 |
| Expansive | I0: 35<br>I1: 19<br>I2: 13<br>I3: 2<br>I4: 1 | |
| **Tumor budding** | | |
| No | I0: 23<br>I1: 8<br>I2: 6<br>I3: 2<br>I4: 1 | 0.217 |
| <10 | I0: 18<br>I1: 16<br>I2: 10<br>I3: 0<br>I4: 0 | |
| 10-19 | I0: 11<br>I1: 2<br>I2: 0<br>I3: 0<br>I4: 0 | |
| >=20 | I0: 5<br>I1: 1<br>I2: 1<br>I3: 0<br>I4: 0 | |
| **Histology** | | |
| Conventional | I0: 24<br>I1: 8<br>I2: 5<br>I3: 1<br>I4: 0 | 0.289 |
| Serrated | I0: 16<br>I1: 10<br>I2: 2<br>I3: 0<br>I4: 0 | |
| Comedo-cribiform | I0: 7<br>I1: 4<br>I2: 4<br>I3: 0<br>I4: 0 | |
| Mucinous | I0: 4<br>I1: 4<br>I2: 1<br>I3: 0<br>I4: 0 | |
| **Serrated adenocarcinoma** | | |
| Yes | I0: 16<br>I1: 10<br>I2: 2<br>I3: 0<br>I4: 0 | 0.337 |
| | I0: 43 | |

| | | |
|---|---|---|
| No | I1: 18<br>I2: 16<br>I3: 2<br>I4: 1 | |
| **Vascular infiltration** | | |
| Yes | I0: 5<br>I1: 4<br>I2: 3<br>I3: 0<br>I4: 0 | 0.814 |
| No | I0: 53<br>I1: 24<br>I2: 15<br>I3: 2<br>I4: 1 | |
| **Lymphatic infiltration** | | |
| Yes | I0: 16<br>I1: 10<br>I2: 4<br>I3: 1<br>I4: 0 | 0.753 |
| No | I0: 42<br>I1: 18<br>I2: 14<br>I3: 1<br>I4: 1 | |
| **Perineural infiltration** | | |
| Yes | I0: 9<br>I1: 4<br>I2: 3<br>I3: 0<br>I4: 0 | 0.964 |
| No | I0: 49<br>I1: 24<br>I2: 15<br>I3: 2<br>I4: 1 | |
| **Macrophages (CD163 spindle-shape)** | | |
| Yes | I0: 33<br>I1: 25<br>I2: 16<br>I3: 2<br>I4: 0 | 0.010 |
| No | I0: 10<br>I1: 0<br>I2: 0<br>I3: 0<br>I4: 0 | |
| **Macrophages (CD163 round)** | | |
| Yes | I0: 17<br>I1: 10<br>I2: 12<br>I3: 0<br>I4: 0 | 0.121 |
| No | I0: 26<br>I1: 15<br>I2: 6<br>I3: 2<br>I4: 0 | |
| **Macrophages (CD163 total)** | | |

| | | |
|---|---|---|
| Positive | I0: 36<br>I1: 25<br>I2: 16<br>I3: 2<br>I4: 0 | 0.055 |
| Negative | I0: 7<br>I1: 0<br>I2: 0<br>I3: 0<br>I4: 0 | |
| **Adjuvant treatment** | | |
| Yes | I0: 24<br>I1: 12<br>I2: 6<br>I3: 2<br>I4: 0 | 0.398 |
| No | I0: 35<br>I1: 16<br>I2: 12<br>I3: 0<br>I4: 1 | |
| **Metastatic chemotherapy regimen** | | |
| Qx alone | I0: 2<br>I1: 0<br>I2: 1<br>I3: 0<br>I4: 0 | |
| Qx + antiEGFR | I0: 3<br>I1: 3<br>I2: 1<br>I3: 0<br>I4: 0 | 0.612 |
| Qx + antiangiogenic | I0: 2<br>I1: 1<br>I2: 2<br>I3: 0<br>I4: 0 | |
| **Number of metastatic locations at diagnosis**<br>**Non- regional nodes** | | |
| Hepatic | I0: 5<br>I1: 2<br>I2: 1<br>I3: 0<br>I4: 0 | 0.299 |
| Peritoneal | I0: 1<br>I1: 2<br>I2: 3<br>I3: 0<br>I4: 0 | |
| Lung | I0: 0<br>I1: 1<br>I2: 0<br>I3: 0<br>I4: 0 | |
| **Number of metastatic locations at progression**<br>**Non-regional nodes** | | |

| | | |
|---|---|---|
| Hepatic | I0: 4<br>I1: 1<br>I2: 2<br>I3: 0<br>I4: 0 | 0.196 |
| Peritoneal | I0: 1<br>I1: 4<br>I2: 2<br>I3: 0<br>I4: 0 | |
| Lung | I0: 6<br>I1: 1<br>I2: 1<br>I3: 0<br>I4: 0 | |
| Bone | I0: 2<br>I1: 0<br>I2: 1<br>I3: 0<br>I4: 0 | |

The reference categories for each variable are as follows: for clinical stage, Stage I; for sidedness, left-sided tumors; for histological grade, G1 (well-differentiated); for tumor budding, no tumor budding; for immunoscore, low immunoscore; for adjuvant treatment, no adjuvant treatment; and for molecular subtypes, CMS1. All *p*-values represent results from chi-square tests or contingency tables unless specified otherwise. *Kruskal-Wallis test.

## **Supplementary Table 3.** Survival analysis using Cox proportional hazards model (univariate).

| | Overall Survival | | | Relapse-free survival | | |
|---|---|---|---|---|---|---|
| | HR* | 95%CI* | *p*-value | HR* | 95%CI* | *p*-value |
| **Sex** | 0.679 | 0.468-0.985 | 0.041 | 0.905 | 0.562-1.457 | 0.681 |
| **ECOG*** | 2.651 | 2.099-3.347 | <0.001 | 1.602 | 1.131-2.268 | 0.008 |
| **T** | 1.416 | 1.069-1.875 | 0.150 | 2.350 | 1.569-3.520 | <0.001 |
| **N** | 1.638 | 1.316-2.039 | <0.001 | 2.434 | 1.811-3.273 | <0.001 |
| **M** | 2.717 | 1.821-4.054 | <0.001 | 4.905 | 3.021-7.965 | <0.001 |
| **Clinical stage** | 1.647 | 1.330-2.040 | <0.001 | 1.451 | 1.289-1.633 | <0.001 |
| **Sidedness** | 0.883 | 0.618-1.261 | 0.493 | 1.000 | 0.624-1.603 | 1.000 |
| **Polyps** | 1.069 | 0.747-1.528 | 0.716 | 0.703 | 0.439-1.127 | 0.144 |
| **CEA (presurgical)** | 1.487 | 0.925-2.388 | 0.101 | 2.102 | 1.211-3.649 | 0.008 |
| **Histological grade** | 1.393 | 1.025-1.893 | 0.034 | 2.136 | 1.431-3.188 | <0.001 |
| **MSI*** | 0.790 | 0.505-1.236 | 0.302 | 0.972 | 0.521-1.814 | 0.930 |
| **CIMP** | 1.126 | 0.872-1.454 | 0.362 | 1.104 | 0.802-1.519 | 0.546 |
| ***BRAF*** | 0.498 | 0.284-0.874 | 0.015 | 1.048 | 0.422-2.603 | 0.920 |
| ***KRAS*** | 0.997 | 0.697-1.426 | 0.987 | 0.916 | 0.573-1.465 | 0.715 |
| ***NRAS*** | 1.622 | 0.893-2.946 | 0.112 | 1.606 | 0.736-3.509 | 0.234 |
| **CDX2 (positive/negative)** | 0.510 | 0.291-0.897 | 0.019 | 0.475 | 0.226-0.999 | 0.049 |
| **Tumor growth pattern** | 1.886 | 1.316-2.702 | 0.001 | 2.851 | 1.765-4.605 | <0.001 |
| **Tumor budding*** | 0.960 | 0.869-1.059 | 0.414 | 0.931 | 0.819-1.059 | 0.278 |
| **Histology** | 1.182 | 1.059-1.319 | 0.003 | 1.072 | 0.927-1.239 | 0.349 |
| **Serrated adenocarcinoma** | 1.528 | 1.032-2.260 | 0.034 | 1.244 | 0.734-2.107 | 0.417 |
| **Vascular infiltration** | 2.466 | 1.626-3.739 | <0.001 | 4.716 | 2.895-7.684 | <0.001 |
| **Lymphatic infiltration** | 2.065 | 1.437-2.967 | <0.001 | 3.251 | 2.029-5.209 | <0.001 |
| **Perineural infiltration** | 1.982 | 1.338-2.937 | 0.001 | 3.286 | 2.034-5.310 | <0.001 |
| **Macrophages (CD163 spindle-shape)** | 0.910 | 0.500-1.656 | 0.758 | 1.151 | 0.486-2.728 | 0.750 |
| **Macrophages (CD163 round)** | 1.008 | 0.643-1.580 | 0.974 | 0.825 | 0.452-1.505 | 0.530 |
| **Macrophages (CD163 total)** | 0.855 | 0.451-1.623 | 0.633 | 1.347 | 0.481-3.770 | 0.571 |
| **Adjuvant treatment** | 0.594 | 0.406-0.867 | 0.007 | 1.212 | 0.758-1.937 | 0.422 |
| **Metastatic chemotherapy regimen** | 1.305 | 0.802-2.121 | 0.284 | 1.155 | 0.714-1.868 | 0.558 |
| **Metastatic location at diagnosis** | 1.124 | 0.930-1.359 | 0.226 | 1.166 | 0.950-1.431 | 0.142 |
| **Metastatic location at progression** | 0.956 | 0.859-1.064 | 0.411 | 0.837 | 0.749-0.936 | 0.002 |

Hazard ratios (HRs), 95% confidence intervals (CIs), and *p*-values were obtained from univariate Cox proportional hazards models. Variables marked with an asterisk (*) showed discrepancies compared to the univariate Breslow (generalized Wilcoxon) test reported in the main Results section. Such differences may occur because the Breslow test gives greater weight to early events, whereas Cox regression assumes proportional hazards over time.

**Supplementary Figure 1**. Forest plot of multivariate Cox analysis for overall survival.

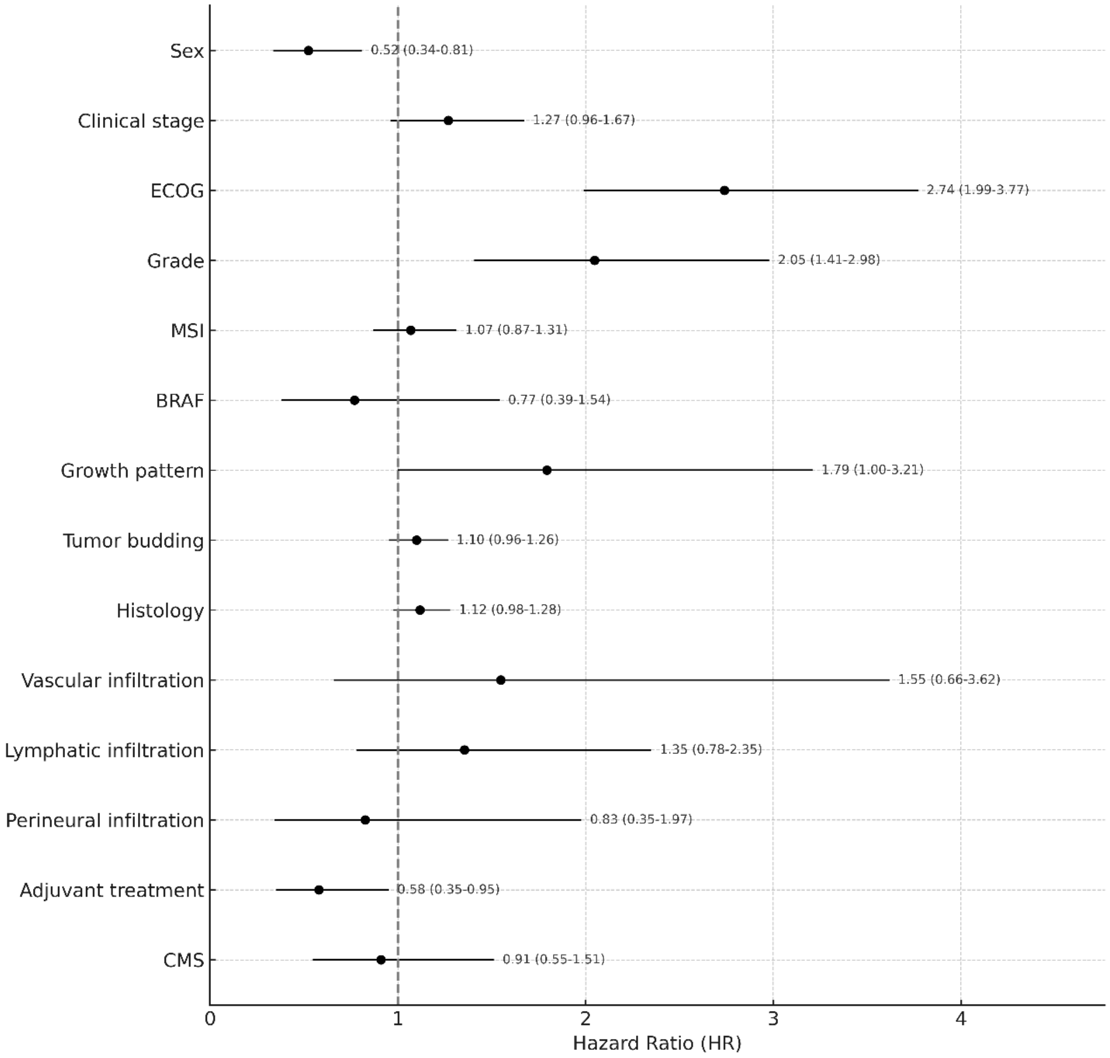


Forest plot showing hazard ratios (HRs) and 95% confidence intervals (CIs) for clinical, pathological, and molecular features associated with overall survival. HRs and CIs were derived from multivariate Cox proportional hazards models. The vertical dashed line indicates HR = 1 (no effect).

**Supplementary Figure 2**. Forest plot of multivariate Cox analysis for relapse-free survival (RFS).

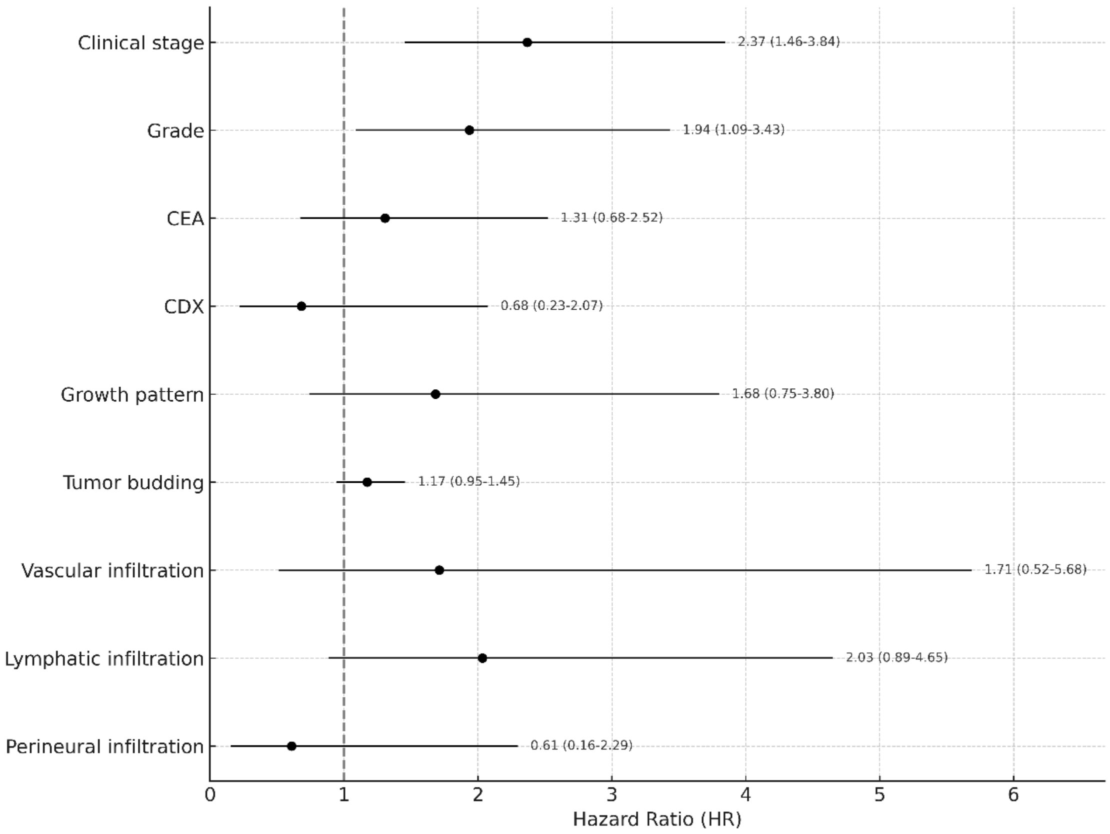


Forest plot showing hazard ratios (HRs) and 95% confidence intervals (CIs) for clinical, pathological, and molecular features associated with relapse-free survival. HRs and CIs were derived from multivariate Cox proportional hazards models. The vertical dashed line indicates HR = 1 (no effect).
